# Can Team Resilience Boost Team Creativity Among Undergraduate Students? A Sequential Mediation Model of Team Creative Efficacy and Team Trust

**DOI:** 10.3389/fpsyg.2021.604692

**Published:** 2021-06-10

**Authors:** Mudan Fan, Wenjing Cai, Lin Jiang

**Affiliations:** ^1^School of Education, Weinan Normal University, Weinan, China; ^2^Intellectual Property Research Institute, University of Science and Technology of China, Hefei, China; ^3^School of Public Affairs, University of Science and Technology of China, Hefei, China; ^4^Department of Management and Organization, Vrije Universiteit Amsterdam, Amsterdam, Netherlands

**Keywords:** team resilience, team efficacy, team creativity, undergraduate students, team trust

## Abstract

Although recent literature has highlighted the critical role of resilience in creativity literature, existing findings have failed to indicate the processes through which resilience contributes to creativity at the graduate level. The current study fills this gap by hypothesizing the influence of team resilience on team creativity through a sequential mediating mechanism. A time lagged research study was conducted, and a sample of 201 undergraduate students and their teacher filled out questionnaires at three different time points (with 2-week intervals). After aggregating the data at the team level, we employed the PROCESS macro in SPSS to analyze data and test all the hypotheses through performing a sequential mediation analysis. We found that (a) team resilience would predict team creativity; and (b) team efficacy and team trust sequentially mediated the relation between team resilience and team creativity. The results in our study advance the emergent literature on linking resilience and creativity for the practical applications of resilience and creativity in education settings.

## Introduction

As a necessity to thriving in the 21st century, creativity has been highlighted in colleges and universities, which have an obligation to help cultivate students’ creativity (Parker-Bell, 2010). In educational settings, creativity represents a student’s way of thinking, learning, and producing information in school courses, such as science and mathematics (Torrance and Goff, 1990), which reflects the characteristic of “problem solving.” Extensive literature has indicated that educators are increasingly focused on developing students’ creativity defined as students producing novel and useful ideas and solutions to address challenges and problems (Amabile, 1997). Specifically, scholars have provided strong evidence indicating that personal factors, such as Big-Five personality traits, are the traits most central to creativity and positive psychology (i.e., PsyCap). Among this line of research, *resilience* is found to play a role in fostering creativity (Kim, 2015; Fernandez-Martinez et al., 2017). Defined as individuals’ ability to bounce back from risks or failures and to adapt to dynamics and success, resilience can ensure students to try to solve problems, exhibit optimism, become positive role models, and show flexibility (Haglund et al., 2007). Previous research has indicated that resilient students have more psychological safety in overcoming the challenges that accompanied creative endeavors (Luthans et al., 2004).

However, an important yet neglected research problem is still unclear—that is, *whether and how resilience contributes to creativity among graduate students at the team level*. Theoretically, team resilience refers to the extent to which a team believes its capabilities on effectively coping tasks and recovering positively to difficulties together (Carmeli et al., 2014). Understanding the intervening processes through which team resilience can contribute to undergraduate students’ creativity is important for effective policy development and intervention implementation in educational settings. First, a recent review indicates that limited empirical studies have been conducted to identify how team resilience helps teams adapt to adversity during creative processes (Chapman et al., 2020). The facts show that it is not only individuals who face difficulties but also teams that commonly experience adversity (Alliger et al., 2015). In educational settings, especially in universities and colleges, students are encouraged to improve their communication and social relationships with other individuals and groups (Urdan and Schoenfelder, 2006; Kim and Kim, 2017); therefore, to respond to scholars’ calling for testing the effect of team resilience on desirable team outcomes (Chapman et al., 2020), examining the association between team resilience and team creativity among undergraduate students is urgently needed for theoretical development and educational practices. Moreover, previous research has indicated the mediating roles of psychological factors such as well-being and personal psychological resources (Richtner and Lofsten, 2014; Arnout and Almoied, 2020). However, the results overlooked the potential mediating role of some key psychological characteristics at the team level and only illustrated the partial mediation models. Thus, exploring the sequence of some team-level psychological mediators becomes an important concern since causal mechanisms can provide a more comprehensive picture to clearly depict the effects of team resilience on team creativity.

**FIGURE 1:**
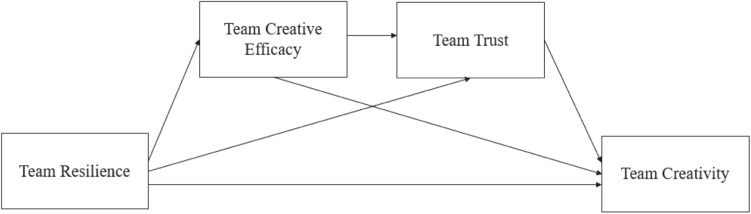
Proposed sequential mediation model.

As such, by inviting undergraduate students to organize temporary teams for a research project, this study examines the potential linkage between team resilience and team creativity via exploring the sequential mediating roles of team creative efficacy and team trust. Specifically, we draw on social identity theory to propose two mediators—i.e., team creative efficacy and team trust. Team creative efficacy refers to team members’ shared belief on their team’s ability of achieving a particular goal (Bandura, 1997), and team trust refers to team members’ shared belief on whether they are free to share both task-related and personal information without any concern for differences. The social identity approach suggests that individuals’ sense of self can be predominately defined in terms of their social identity (i.e., their sense of themselves as group members who share goals, values, and interests with others) (Tajfel et al., 1979). Previous studies applying this theoretical framework have indicated that team members whose sense of self is as group members (as “we” and “us”) have more positive psychological characteristics (e.g., attachment) (Cameron, 1999; Postmes and Branscombe, 2010) toward making more contributions to the group. By following this line of study, we expect team creative efficacy and team trust to be two prominent mediators; that is, when team resilience is high, team members are more likely to build their creative efficacy belief on behalf of their own teams, which in turn effectively fosters the team. Figure 1 shows our proposed sequential mediation model.

### Team Resilience

In the area of resilience research, some studies specifically examined the resilience among students (Kim, 2015; Fernandez-Martinez et al., 2017), because high levels of stress and related academic burnout are widespread among graduate students (Dyrbye et al., 2010; Divaris et al., 2012). Specifically, resiliency has been suggested as a mediator to the stressors of learning and may have positive long-term and far-reaching effects among students (Johnson, 2008). In the educational settings, a student’s resilience can be characterized as “the capacity to resist or manage adversity without developing physical or psychological disabilities” during school training (Campbell-Sills et al., 2006). Recently, scholars’ attention is increasingly transferring to the team level phenomena by investigating team resilience (Chapman et al., 2020), because both individuals and groups have to face difficulties and experience adversity (Bowers et al., 2017). Theoretically, by representing the critical team level capacity that facilitates the rebound of teams after an adverse event, team resilience can be defined as “a team’s belief that it can absorb and cope with strain, as well as a team’s capacity to cope, recover and adjust positively to difficulties” (Carmeli et al., 2014, p. 149). Teams that thrive, rebound, or positively adapt to adversity are more unlikely to experience the deleterious effects of challenging situations. Through examining resilience at the team level, researchers attempt to identify how teams and groups positively adapt to adversity (Bennett, 2010; Alliger et al., 2015; Consoli et al., 2015).

In the educational context, existing research evidence has confirmed that students who develop resilience are better equipped to learn from failure and adapt to change (Yeager and Dweck, 2012); thus resilient teams should be more flexible to adverse changes and well prepared for future planning and preparation (e.g., Cavrak et al., 2019). Relating to the participants in the current study, namely, undergraduate students majoring in hospitality management, hospitality professional education is perceived by many students to be a stressful experience with students studying service-related courses reporting increased levels of anxiety, fatigue, burnout and lack of motivation. Therefore, resilient students are more likely to cope with such adversities and achieve better results (Kwek et al., 2013; Jones and Wynn, 2019). In addition, team-based learning has become a prominent trend in hospitality-related courses (Jacobs et al., 2001). Therefore, faced with such challenges and requirements, it is important to investigate the phenomena and the effects of team resilience among undergraduate students by studying the associations of team resilience with other desirable outputs in educational settings.

### Team Creativity

Creativity has been highlighted in the educational context by showing its merits of developing students’ potential to address unexpected challenges by coming up with creative solutions (Torrance and Goff, 1990). Targeting solving problems, researchers and educators in the hospitality and service fields are calling for the development of students’ creativity due to the increased competition in this industry (Liu et al., 2017). Consistent with this line of research, scholars have recently investigated students’ creativity at the team level (Rego et al., 2007; Aggarwal and Woolley, 2018; Bodla et al., 2018) since creative activities in teams can solve problems and leverage opportunities through the integration of divergent thoughts and perspectives (Barczak et al., 2010).

Given that an individual’s creative inputs might not directly contribute to the whole team’s creative achievements, to understand the factors that drive team creativity (Kurtzberg and Amabile, 2000), it is important to extend the focus of analysis from the creative capabilities of the individual team members to team interaction processes and emergent states. Researchers conducting studies among students have consistently found that some contextual factors, especially positive team-oriented variables (e.g., team diversity), can predict team creativity (Grawitch et al., 2003; Kim et al., 2007; Curseu, 2010). For example, Barczak et al. (2010) found that members’ perceptions that their peers are reliable and competent are vital to enhancing the creativity of the team.

### Team Rresilience and Team Creativity

Previous studies have accumulated evidence by revealing that team resilience is beneficial to some desirable outcomes. Based on these findings, in the current study, we expect to discover a positive relation between team resilience and team creativity. Specifically, when students are learning in resilient teams, they could feel that their teams are displaying an ability to thrive in situations of adversity, improvise and adapt to significant change or stress. In this situation, they may be unlikely to experience the potentially damaging effects of threatening situations. As a result, the teams’ potential to engage in creative endeavors to realize creative ideas will be high. Moreover, Waxman et al. (2003) have consistently shown that a high level of resilience enables students to maintain high motivational achievement and performance, even when they are faced with stressful events and conditions that place them at risk of poor performance.

### Social Identity Theory

Social identity theory is a psychologically oriented theory that indicates that individuals gain part of their self-concept from memberships in social groups (Tajfel, 1978). Specifically, social identity is a part of an individual’s self-concept that originates from his membership of a social group together with the value and emotional significance attached to that membership (Tajfel, 1978, p. 63). Previous literature drawing on this theory has illustrated that individuals recognize their own membership in groups by defining the social boundaries surrounding particular groups and then self-categorizing themselves as either belonging or not belonging to those groups (Postmes and Branscombe, 2010).

Social identity theory has been widely used in the educational literature to understand students’ desirable outcomes (e.g., learning in context) (e.g., Kelly, 2009). Specifically, both educational psychologists (adopting a social identity perspective) and social psychologists (applying the social identity approach to educational settings) focus on the influence of social identities on various aspects of learning-related behaviors and/or attitudes among students (e.g., Bliuc et al., 2011). For example, Edwards and Harwood (2003) found that students’ social identification is related to perceptions of favored and disfavored instructors.

### The Mediator of Team Creative Efficacy

Team creative efficacy is a team-level concept that is defined as a shared belief concerning a team’s ability to organize and execute courses of action required to achieve a specific outcome (Bandura, 1997). In the educational context, team creative efficacy among students represents a shared belief in collaborating to develop the creativity of the process during collaborative learning activities (Cheng and Yang, 2011). Previous studies have shown that creative efficacy belief is a beneficial type of personal psychological state that contributes to facilitating students’ desirable outcomes regarding creativity (e.g., Fan and Cai, 2020). Relatedly, team creative efficacy specifically representing a team’s psychological state has been outlined by the bulk of the literature which indicates that when a team is characterized by high resilience, team members are significantly motivated to produce positive achievements. For example, in Lyons et al. (2016) qualitative study, they found that when students expressed confidence in their collective efficacy, they were more likely to act as a collective agency toward such behaviors as solving problems together and attending to relationships. Moreover, there is a prominent research stream underlining the beneficial role of team creative efficacy on individuals’ engagement in team creative processes (Shin and Zhou, 2007) because all team members share a high level of confidence in their joint efforts to come up with creative solutions. For example, empirical work by Shin and Eom (2014) shows that teams with high creative efficacy are more likely to achieve higher levels of team creativity than teams with low creative efficacy.

According to the theoretical suggestion of social identity theory, team resilience can strengthen all team members’ identification with their group because this team-level phenomenon represents a specific psychosocial phenomenon, and the collective psychological state of team members’ common cognition, motivation and emotion is triggered (Kennedy et al., 2017). In this situation, team members raise a sense of “us” and treat their own efforts as an important contribution to the whole team. Furthermore, the higher level of resilience the team obtains, the greater the group membership that will be experienced by all the team members. Team resilience may generate more team-oriented attribution. Following this line of reasoning, it is reasonable to expect a positive relation between team resilience and team creative efficacy. Specifically, researchers have indicated that teams that encompass a broader perspective in the face of adversity tend to develop a positive adaption (Bennett, 2010).

### The Mediator of Team Trust

Team trust is a psychological state comprising the intention to accept vulnerability based upon positive expectations of the intentions or behavior of another. This interpersonal attribute is one of the important elements of teamwork and is based on both emotional bonds and perceived competencies of individual members (Barczak et al., 2010). When members trust each other, they tend to feel less vulnerable, which facilitates the channeling of energy for creating and discovering rather than defending (Gibb, 1978). In educational settings, scholars and educators acknowledge that building trusting relationships with team members plays a crucial role in learning development and knowledge creation (Tseng and Yeh, 2013). For example, given that trust stresses interpersonal and interdependent group dynamics, when learners perceive team trust during their study period, the effectiveness of their online learning teams increases significantly (Chen et al., 2011; Deortentiis et al., 2013).

Based on the theoretical framework of the social identity approach, as resilience at the team highlights the individual’s sense of “us” within the team, when teams are characterized as resilient, all the team members tend to display such behaviors on behalf of their teams as effective collective actions in the face of highly complex environmental conditions (Hambrick, 1994). This happens because resilient environments in the team facilitate team members’ connections with each other, in terms of identity (Roberts, 2007). Consequently, they (i.e., team members) develop positive relationships—e.g., trust—based on their sense of security to express their true feelings (Stephens et al., 2013).

Previous creativity literature has suggested the benefits of team trust on team creative outputs (Kipkosgei et al., 2020). Generally, trust is identified as a critical feature for promoting successful partnerships among diverse members of a team, because trust is key to holding members together as a cohesive unit (Kasper-Fuehrera and Ashkanasy, 2001; Bijlsma and Koopman, 2003). Since creative teams are known for their ability to identify and exploit unique opportunities by using imaginative strategies to procure and orchestrate resources across functional groups (Cheng, 2011), team trust supports better communication, information sharing, focus and greater cooperation (Barczak et al., 2010).

## Overview of the Current Study

The above review and reasoning establish that resilience contributes to undergraduate students’ creativity at the team level. However, more empirical examinations are required in the creativity literature to explore the processes by which team resilience contributes to team creativity among undergraduate students. Based on the abovementioned discussion, we draw on social identity theory to expect the potential serial mediation effects of team creative efficacy and team trust.

First, we assess the potential positive association between team resilience and team creativity. Specifically, as resilient teams should be more flexible to adverse changes, it is reasonable to predict that teams with a high level of resilience tend to generate more flexible and adaptive responses to adversity (Meneghel et al., 2016); additionally, they are more likely to use setbacks as challenges or opportunities for coming up with creative solutions (Carmeli et al., 2014). Thus, we hypothesize a positive relationship between team resilience and team creativity. That is, team resilience is positively related to team creativity (H1).

Second, we examine the mediating roles of team creative efficacy in linking team resilience and team creativity among undergraduate students. Specifically, according to the theoretical arguments in social identity theory, when studying in a team characterized as highly resilient, team members view their teams as having the capacity for positive adaptation through collective interactions (Bowers et al., 2017). Thus, team members tend to build a strong sense of confidence about their teams’ capability to address creative problems. In this situation where all students share a high level of confidence in their joint efforts within the team, they are more likely to come up with creative solutions by working together. Thus, we propose the next hypothesis: team creative efficacy mediates the relationship between team resilience and team creativity (H2).

Third, we examine the other mediator—i.e., team trust—linking the positive association between team resilience and team creativity. Specifically, resilient teams in the face of adversity are more likely than non-resilient teams to increase all members’ attentiveness within the team toward building team trust. In this trusting environment, team members are more willing to take a risk by sharing information and cooperating with their team members (Mayer et al., 1995), resulting in a creative solution to their task. Accordingly, we propose that team trust mediates the relationship between team resilience and team creativity (H3).

Finally, we explore a sequential mediating process to address the following question: How do team creative efficacy and team trust relate to each other in the social context and relate to the process of creative performance at the team level? Specifically, in teams with a high level of resilience, team members may identify themselves with the whole team by developing their joint efforts in a creative manner, thereby increasing the teams’ creative performance. That is, team resilience can directly enhance all the members’ sense of the teams’ confidence in being creative, thus providing a sound working environment of trust in the teams, which finally facilitates the teams’ creative outputs. In addition, as social identity theory suggests, in the team process, team members’ social identity points to particular social psychological processes—that is, one member’s psychological state can transfer to other team members. Regarding collective efficacy belief, team creative efficacy—representing team members’ shared belief regarding the team’s ability to accomplish a creative task—may result in building trust within a team, because individuals holding greater beliefs about their teams’ creative capabilities may reinforce more interactive activities with other team members; thus, these members tend to develop a sense of trust with other members within the team. Therefore, we propose the final hypothesis that team creative efficacy and team trust sequentially mediate the relationship between team resilience and team creativity (H4).

## Materials and Methods

### Procedure and Participants

The sample in the current study was composed of 201 undergraduate students from a university in mainland China. This university was chosen from the collaborating members in our research project which aims to explore the predictors of students’ creativity in Chinese universities. At this university, courses were designed to stimulate learners’ creativity, and undergraduate students participated in creative activities in and after class. Among all the departments in this university, we randomly selected the Department of Hospitality Management to participate in our research. One of the authors contacted the teacher from the department of hospitality management to confirm whether she would like to join our research project with her students. After receiving her confirmation, we started our survey research in the teacher’s course. We decided to involve students who were enrolled in a second-year bachelor’s course on hospitality management. These students not only accumulated related knowledge about the hospitality and tourism industry but also got along with their classmates after the first year of study; thus, they could work closely to complete a class project by collectively initiating creative tasks in hospitality-related business topics. These students were informed that their participation helped them fulfill a course requirement and obtain course credits. To guarantee confidentiality, all participants involved were informed of the survey objectives at the very beginning of the study. The teacher asked all the undergraduate students to complete the paper-and-pencil questionnaires in the classroom during the class period. When they completed the survey, they returned it directly to the teacher’s hands. Afterward, the teacher sent the questionnaires to the author.

The teacher initiated a project that developed marketing plans in the modern hospitality industry. In this project, students should provide a final proposal including the real-world marketing policies and in-depth analysis of some hospitality managerial issues. All the undergraduate students were involved in completing this project by working with a team. That is, they were asked to organize teams by themselves, and each team had 5–8 team members. Before forming project teams, members were asked to work closely with their teammates to complete their projects during this project by researching and discussing information, such as customer profiles, the marketing environment and competition, which are required for the project.

A 1-month milestone agenda was suggested to the teams. In the first week, student participants organized their own team and initiated some project plans. During this week, 31 teams were formed, and team members were getting close to each other within each team. After forming teams, we started our time-lagged research design in the following weeks. Specifically, at Time 1, undergraduate students were asked to rate their team resilience. After 1 week, at Time 2, undergraduate students were asked to rate their team efficacy and their team trust. After 1 week, at Time 3, the teacher was asked to rate each team’s creativity. Among these student participants (*N* = 201), 66.2% were male (*SD* = 0.47), and the average number of team members in each team was 6.48.

### Measurements

We used validated scales from previous literature. Since these scales are originated and developed in papers written in English, these English original scales are required to be translated to have an accurate and high quality questionnaire. The back-translation method was employed to provide a Chinese instrument (Brislin, 1986). Seven-point Likert scales (from 1 = strongly disagree, to 6 = strongly agree) were used.

#### Team Resilience

A seven-item scale from Mallak (1998) was used to assess resilience at the team level (Cronbach’s α = 0.89) which refers to a team’s collective resources can be harnessed to positively adapt to adversity. The original scale shows good reliability (Cronbach’s α from 0.85 to 0.95) in previous studies. Our questionnaire asked students to rate the extent to which their team has the capacity to bounce back from failure, setbacks, conflicts, or any other threat to well-being. One sample item is “In difficult situations, my team tries to look on the positive side.” The Kaiser–Meyer–Olkin (KMO) value was 0.88, with the Bartlett test of sphericity achieving statistical significance (*p* < 0.001).

#### Team Creative Efficacy

We adopted the four-item scale from Shin and Eom (2014) to measure team creative efficacy belief (Cronbach’s α = 0.87) which refers to team members’ shared beliefs in their team’s capabilities to generate creative ideas together. This scale has been widely used in prior studies which generate good reliability (Cronbach’s α from 0.80 to 0.93). Since we specifically examined the influences of team green-oriented efficacy belief, we designed these items to explicitly represent the team members’ shared beliefs in their team’s capabilities of performing green innovative tasks. One sample item is “Our team is able to solve green tasks if we invest the necessary effort.” The KMO value was 0.79, with the Bartlett test of sphericity achieving statistical significance (*p* < 0.001).

#### Team Trust

We used the four-item scale from Bierly et al. (2009) (Cronbach’s α = 0.82) to rate team trust referring to team members’ willingness to rely on each other to take accountability as a whole team. The validity of this scale has been shown in previous studies (Cronbach’s α from 0.84 to 0.89). We asked undergraduate students to assess their own teams’ trust. One sample item is “Over-all, the people on my team were very trustworthy.” The KMO value was 0.75, with the Bartlett test of sphericity achieving statistical significance (*p* < 0.001).

#### Team Creativity

We used the eight-item scale from Rego et al. (2007) (Cronbach’s α = 0.92) to rate team creativity referring to teams producing novel ideas and solutions to address challenges and problems. This is a widely used scale in the educational literature during to its high validity (Cronbach’s α from 0.81 to 0.95). We asked the teacher to assess each team’s creativity based on team’s final proposals. One sample item is “Team members come up with creative solutions to problems.” The KMO value was 0.88, with the Bartlett test of sphericity achieving statistical significance (*p* < 0.001).

#### Control Variables

We control the team size (i.e., the number of team members) as past literature suggested its potential influence on creative outcomes at the team level (Barczak et al., 2010).

### Analytical Strategy

We first aggregated data from the individual to the team level. Because team resilience, team efficacy, and team trust all represent the shared perception of the team members’ belief and attitude, the team members’ (i.e., undergraduate students’) responses to these team-level characteristics were aggregated to form a measure at the team level. We computed *r*_*wg*_ to evaluate the interrater agreement, ICC(1) (intraclass correlation coefficient) to evaluate the intraclass correlations, and ICC(2) to evaluate the reliability of the group means (Bliese, 2000). The team resilience results indicated that ICC(1) is 0.11, ICC(2) is 0.58, and the average *r*_*wg*_ is 0.86. The team efficacy results showed that ICC(1) is 0.13, ICC(2) is 0.54, and the average *r*_*wg*_ is 0.85. The team trust results showed that ICC(1) is 0.10, ICC(2) is 0.51, and the average *r*_*wg*_ is 0.83. All these indicators show that our data aggregation is appropriate.

Before testing hypotheses, we first used the SPSS software version 21 (Chicago, IL, United States) to analyze the data. Specifically, we calculated the descriptive statistics to characterize all the variables in the current study—computing Pearson’s product-moment correlation to test the directions and correlations among all the variables. To test our hypothesis that team creative efficacy and team trust act as serial mediators of the relationship between team resilience and team creativity, we used the SPSS PROCESS macro, Model 6, to test the stability and significance of the mediation effects. Particularly, we calculated 95% confidence intervals of the indirect effects derived from bias-corrected bootstrap estimates with 5,000 iterations, which are significant at *p* = 0.05 if the 95% confidence interval does not include zero. We employed PROCESS to test our hypotheses because it is widely used in the social, business, and health sciences to estimate direct and indirect effects in single and multiple mediation models (e.g., Hayes and Scharkow, 2013; Baroudi et al., 2018). PROCESS generates all of the statistics calculations and implements bootstrapping in a way that facilitates inference about moderated and mediated effects (Hayes and Scharkow, 2013; Hayes et al., 2017). In the current study, specifically, we used the Model 6 to perform a sequential mediation analysis which explicitly test how the independent variable (i.e., team resilience) can influence the dependent variable (i.e., team creativity) through influencing two distinguished mediators in a sequential way (i.e., influencing team efficacy and then team trust).

## Results

### Descriptive Analysis

We present the descriptive statistics of the variables in Table 1. The results show that team resilience is significantly correlated with team creativity (β = 0.23, *p* < 0.05), and the correlation coefficient presents the expected positive significance, providing initial support for H1. As discussed, team resilience also correlates to team creative efficacy (β = 0.39, *p* < 0.05) and team trust (β = 0.45, *p* < 0.05). Moreover, both team creative efficacy (β = 0.50, *p* < 0.05) and team trust (β = 0.37, *p* < 0.05) correlate to team creativity. The results are consistent with our expectations.

**TABLE 1:** Descriptive statistics and correlations between variables.

Variables	Mean	*SD*	1	2	3	4
(1) Team size	6.32	0.98				
(2) Team resilience	4.64	0.41	0.17			
(3) Team creative efficacy	4.79	0.40	0.15	0.39**		
(4) Team trust	5.00	0.35	0.09	0.45**	48**	
(5) Team creativity	4.43	0.87	0.31	0.23**	0.50**	37**

N = 31 (team-level). **p < 0.05.

### Confirmatory Factor Analysis and Validity

In order to validate the developed constructs, a measurement model was estimated with a confirmatory factor analysis in which each measurement item was loaded on its proposed constructs, and the constructs were allowed to be correlated in the analysis (Anderson and Gerbing, 1988). All measurement items were loaded on their expected constructs (Table 2). The model indices indicated good fit: χ^2^ = 312.70, df = 153, χ^2^/df = 2.04, RMSEA = 0.07, and SRMR = 0.07, CFI = 0.95, TLI = 0.94.

**TABLE 2:** Results of confirmatory factor analysis and correlations of constructs.

Construct	Standardized factor loadings	Composite reliabilities	AVE	1	2	3	4
(1).Team creative efficacy		0.87	0.64	1			
EFFIC1	0.75						
EFFIC2	0.82						
EFFIC3	0.90						
EFFIC4	0.71						
(2) Team trust		0.78	0.50	0.126***	1		
TRUST1	0.83						
TRUST2	0.72						
TRUST3	0.48						
TRUST4	0.69						
(3) Team resilience		0.89	0.55	0.102***	0.099***	1	
TR1	0.74						
TR2	0.82						
TR3	0.81						
TR4	0.70						
TR5	0.86						
TR6	0.87						
TR7	0.75						
(4) Team creativity		0.95	0.79	0.007	0.045***	0.031**	1
CREA1	0.83						
CREA2	0.91						
CREA3	0.94						
CREA4	0.96						
CREA5	0.80						

AVE, average variance extracted. **p < 0.01, **p < 0.001.

Furthermore, we assessed the composite reliabilities and construct validity. The composite reliability of indicators needed to exceed the cut-off value of 0.70 (Hair et al., 1998). Next, we calculated the average variance extracted (AVE) to check the convergent validity of the constructs. Theoretically, AVE > 0.50 does convey sufficient variance for the variables to converge into a single construct (Hair et al., 1998). The discriminant validity of constructs was assessed when the AVE was compared to the squared correlation between latent constructs; and the squares correlations between constructs were less than the AVE, suggesting discriminant validity (Fornell and Larcker, 1981). The results shown in Table 2 indicated that the AVE of each construct was more than 0.50, composite reliability of indicators was more than 0.70, and the AVE of each construct was higher than the squared correlations between pairs of constructs, indicating construct validity.

### Hypotheses Testing

**FIGURE 2:**
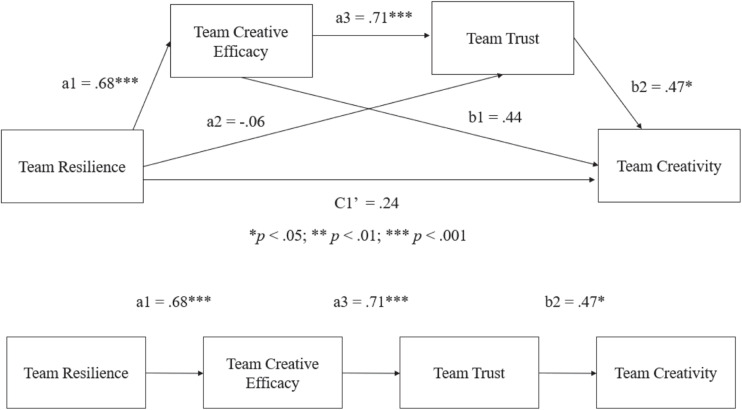
The results of the sequential model with path coefficients.

To test the hypothesis of whether team creative efficacy and team trust sequentially mediate the impact of team resilience on team creativity, we performed a sequential mediation analysis (Model 6, as described in PROCESS) with bootstrap methods (Hayes, 2013). Figure 2 describes all the paths for the full process model. Table 3 displayed the coefficients. The results show that the total effect (C1) of team resilience on team creativity was found to be significant (β = 0.92, *t* = 2.81, *p* < 0.001), supporting H1. However, the results in Table 3 show that the total direct effect (C1’) without the effect of the two mediators was non-significant (β = −0.57, *t* = −1.37, *p* = 0.18). The total indirect effect (i.e., the sum of the specific indirect effects) was significant, with a total indirect effect (β = 0.92, *SE* = 0.32) and a 95% confidence interval between 0.34 and 1.64.

**TABLE 3:** Results of sequential mediation analyses (PROCESS Model 6 in SPSS).

Model 6 Y = Team creativity X = Team resilience M1 = Team creative efficacy M2 = Team trust Sample size: 31 teams								

**Outcome: Model 1:**	**Team creative efficacy**							
	
	**Summary**							

	***R***	***R*-sq**		***F***	**Df1**	**Df2**		***p***
	
	0.70	0.49		13.18	2.00	28.00		0.0001

	**Coefficient**		***SE***		***t***		***p***	

Constant	1.58		0.67		2.36		0.0254	
Team resilience	0.68		0.13		5.02		0.0000	

**Outcome: Model 2:**	**Team trust**							
	
	**Summary**							

	***R***	***R*-sq**		***F***	**Df1**	**Df2**		***p***
	
	0.77	0.59		13.11	3.00	27.00		0.0000

	**Coefficient**		***SE***		***t***		***p***	

Constant	1.89		0.57		3.32		0.0026	
Team resilience	−0.06		0.15		−0.41		0.6819	
Team creative Efficacy	0.71		0.15		4.79		0.0001	

**Outcome: Model 3:**	**Team creativity**							
	
	**Summary**							

	***R***	***R*-sq**		***F***	**Df1**	**Df2**		***p***
	
	0.71	0.50		6.50	4.00	26.00		0.0009

	**Coefficient**		***SE***		***t***		***p***	

Constant	−3.90		1.93		−2.02		0.0535	
Team resilience	−0.57		0.41		−1.37		0.1827	
Team creative efficacy	0.44		0.57		0.78		0.45	
Team trust	0.47		0.55		2.69		0.0124	

**Outcome: Model 4:**	**Team ceativity**							
	
	**Summary**							

	***R***	***R*-sq**		***F***	**Df1**	**Df2**		***p***
	
	0.56	0.39		8.85	3.00	25.00		0.0000

	**Coefficient**		***SE***		***t***		***p***	

Constant	4.34		0.36		3.54		0.0000	
Team resilience	0.92		0.47		2.81		0.0000	

**Total, direct, indirect effects**								
**Total effects of team resilience on team creativity**								

	**Effect**		***SE***		***t***		***p***	

	0.92		0.32		2.81		0.0000	

	**Effect**		***SE***		***t***		***p***	

	−0.57		0.41		−1.37		0.1827	

**Indirect effects of team resilience on team creativity**								

	**Effect**		**Boot *SE***		**BootLLCI**		**BootULCI**	

Total:	0.92		0.32		0.34		1.64	
Ind 1:	0.30		0.39		−0.48		1.12	
Ind 2:	−0.09		0.25		−0.58		0.47	
Ind 3:	0.71		0.40		0.01		1.62	

**Indirect effect key**								

Ind 1:	Team resilience → team creative efficacy → team creativity							
Ind 2:	Team resilience → team trust → team creativity							
Ind 3:	Team resilience → team creative efficacy → team trust → team creativity							

*Analysis notes.*

*Bootstrap samples for bias corrected bootstrap confidence intervals: 5,000.*

*Level of confidence for all confidence intervals in output: 95%.*

*BootLLCI = lower limit confidence interval, BOOTULCI = upper limit confidence interval.*

Moreover, the specific indirect effect resulting from team creative efficacy only was not significant (a1b1 = 0.30; 95% CI = −0.48 and 1.12); and the specific indirect effect resulting from team trust was non-significant (a2b2 = −0.09; 95% CI = −0.58 and 0.47). The results indicated that neither H2 nor H3 are supported.

To test the sequential multiple mediation effect (i.e., H4), the results showed that the specific indirect effect of team resilience on team creativity through both team creative efficacy and team trust (a1a3b2) was significant, with a point estimate of 0.71 and a 95% confidence interval between 0.01 and 1.62, providing full support for H4. Therefore, our proposition—i.e., team resilience is a unique aspect that might lead to positive team creative efficacy, which in turn might be a unique predictor to increase the level of team trust, and the team trust uniquely enhances team creativity—was supported fully by the statistical analysis carried out in the current study. Taken together, the results prove that team creative efficacy and team trust sequentially mediates the linkage between team resilience and team creativity.

## Discussion

### Overview of Findings

Although previous studies examined the potential association between resilience and creativity, limited studies have explored the mediating process on this association at the team level in the educational settings. Focusing on the context of undergraduate students, our results established the positive effect of team resilience on team creativity among undergraduate students. Moreover, we found that team resilience yields better team creativity through higher levels of team creative efficacy and higher team trust; that is, the indirect effect of team resilience on the undergraduates’ team creativity works first through team creative efficacy and then through team trust.

### Theoretical Implications

Our study fills a theoretical void in the literature by linking resilience and creativity at the team level in educational settings. First, we focus on the link at the team level by proposing the positive association between team resilience and team creativity; therefore, we extend the current understanding of the resilience-creativity linkage, from the individual level to the team level. Consistent with previous research findings suggesting that resilient individuals are more likely to behave in a creative way in the workplace setting (Kim, 2015; Fernandez-Martinez et al., 2017), our findings extend this line of thinking by showing that resilience positively relates to creativity among undergraduate students (Waxman et al., 2003; Consoli et al., 2015). By revealing the potential positive linkage between resilience and creativity among undergraduate students in China, we extend current understanding in the educational literature that such students’ positive psychological states as resilience is critical for effective creative work.

At the same time, we used aggregated scores for a team-level analysis, and our results reveal that teams with a high level of resilience can produce more creative outputs. That is, in the situation where undergraduate students organize a team for a project, the team with a high level of resilience is more likely than the team with a low level of resilience to use setbacks as challenges or opportunities for growth (Carmeli et al., 2014); as a result, the team as a whole can come up with more creative responses to adversity. In doing so, we highlight the team resilience as a significant predictor contributes to undergraduates’ collective creativity in the context of higher education; that is, when undergraduates organize a team with a high level of resilience, they can study together toward addressing tasks and projects in a creative manner. This finding specifically suggests that resilient teams experience a greater ability to cope with setbacks and obstacles encountered in the learning and educational context, which in turn allows them overcome adversity and maintain or enhance creative outcomes. These results highlight the need for future research to consider a wider range of perspectives to link undergraduates’ resilience and creativity at team level. For example, according to the theoretical framework of self-regulation process, students teams composed of undergraduates with high resilience may be motivated to regulate their collective behaviors to achieve better outcomes (e.g., creative results).

Moreover, our findings suggest the mediating role of team creative efficacy and team trust in the relationship between team resilience and team creativity. In doing so, we address scholars’ call for exploring the mechanism through which resilience exerts influences on creativity (Bowers et al., 2017). That is, although previous studies have acknowledged that students can self-regulate their psychological factors (e.g., efficacy belief and motivations) to behave creatively (e.g., Gu et al., 2017), existing research failed to empirically uncover the important role of psychological attributes among undergraduate students in the creativity domain. Specifically, we found the sequence of two important psychological factors—i.e., team creative efficacy and tea trust—that link the between team resilience and team creativity. These findings consistently supported the arguments that when students receive such positive information as team resilience and encouragements from their learning contexts, there are more likely to experience positive psychological arousal by developing confidence and interactions within their learning group (Urdan and Schoenfelder, 2006; Tseng and Yeh, 2013; Lyons et al., 2016), which in turn facilitates their creative outputs (e.g., thinking creatively and coming up with creative solutions) (Barczak et al., 2010; Curseu, 2010).

In addition, the serial mediation model offers new insights to the literature by revealing the possibilities of different pathways in explaining the relationship between resilience and creativity at the team level. That is, there is a significant indirect relationship between team resilience and team creativity through both team creative efficacy belief and the level of team trust. In this vein, we empirically demonstrate the intervening processes of psychological flourishing at the team level in linking resilience and creativity in sequence. These findings also suggest the potential “developing” functions of collective confidence and the subsequent potential “building” function of trust within groups through the positive association between team resilience and team creativity, which corroborates the results of earlier studies suggesting the sequential mediators in investigating students; creativity (Miron-Spektor and Beenen, 2015). Since the psychological perspective include a wide range of psychological attributes at the teal level, the complex intervening mechanism requires further research to identify alternative psychology-oriented factors.

Further, through applying social identity theory, we extend the current understanding to better explain the relationship between resilience and creative outcomes at the team level among graduate students. Specifically, previous research primarily employs the emotional and cognitive perspectives to reveal the association between resilience and creativity, which overlooks the collective attributions in the processes (e.g., Bowers et al., 2017; Chapman et al., 2020). However, to address this research limitation, we are among the first attempts to utilize the social identity approach to investigate the psychology and behavior of team members in resilience and creativity literature. In this way, the social identity approach points to particular sequential psychological mechanisms through which team resilience transfers to the team creative outcomes in the educational context (e.g., Haslam et al., 2013). That is, resilient teams transfer to team members by means of team processes that strengthen team members’ collective sense of ‘us,’ as manifested by their increased team creative efficacy beliefs about their creative capabilities, and then enhanced trust among all the team members. Accordingly, our results specifically contribute to developing a social identity approach to students’ creativity that provides a theoretical lens of identity in social environment for integrating and building upon insights provided by established approaches. Meanwhile, we also enrich a core insight of the social identity approach through highlighting some core aspects of identification-oriented process by systematically theorizing about the interactive relationship between the group’s psychological characteristics. In this vein, our findings move beyond relatively vague references to the importance of “team factors” as a mediator between these elements (Nieuwenhuis et al., 2019). To further explore the interplay between learning, identity, and context in the educational context, relevant research in the future could investigate the role of broader social and psychological factors in creative learning among students.

### Educational Implications

Our empirical findings reveal several practical implications for educators. First, building up resilience could help students find creative ways for dealing with their unique difficulties and problems. Given the significant role of team resilience in achieving team creativity, students should be encouraged to develop their internal factors related to resilience, such as optimism and flexibility. For example, teachers can focus praise on students’ efforts for creative thinking and activities. Meanwhile, our findings again imply that relationships are key to team resiliency, and teachers should build a community to help students all become connected to one another. In addition, undergraduate students are encouraged to set and achieve goals through building the practice of self-monitoring, and as a result, they would see the results of their creative work.

Moreover, given team creative efficacy as a key mediator, students can emphasize shaping team member interactions and try to create a communication environment in the teams. For example, team leaders should plan various activities that increase opportunities for member interaction, communication and collaboration. Finally, undergraduate students are encouraged to focus on building trust during their teamwork processes, since team trust is key mediator to transfer the benefits of resilience to creativity. For example, students can organize open communication to build trust in their teams. Meanwhile, teachers can give students more responsibilities to complete their team work; in this way, they would build trust with their teammates toward an increase in team productivity. Finally, since classroom dynamics and teaching methods can shape a classroom culture of resiliency, schools are encouraged to train teachers to reward students when they (i.e., students) obtain good grades or behave in an expected way of being resilience together.

### Limitations and Avenues for Further Research

The present study has some limitations. First, the sample was restricted to Chinese undergraduate students who only majored in hospitality management; therefore, whether the results are applicable to other samples is not confirmed. Future studies are highly encouraged to use other samples to replicate and generalize our findings, such as undergraduate students from science majors. Second, our time-lagged research design was conducted with only 1-week intervals, and thus, we cannot determine causal association for the most part. For example, if the team can provide more creative outputs, all the team members may develop a higher level of resilience (Chen et al., 2018). Accordingly, research in the future can use a longitudinal research design or an experimental research design to re-establish our findings in terms of causality.

Furthermore, according to the theoretical arguments of the social identity theory, contextual factors are likely to stimulate individuals’ specific identity toward a specific outcome, we encourage future research to explore the potential mediators of students’ identity which could transfer the effect of team resilience and team creativity. Taking creative identity role as an example, when team resilience is high, students tend to actively engage in taking risks during their learning processes; as a result, their creative outputs via working together would be higher. The final limitation is about the instruments designed with a 6-point Likert-type scale. Although previous research has indicated that 6-point and 5-point formats are both acceptable for survey studies (Chyung et al., 2017), further studies are still encouraged to use 5- point or 7-point Likert-type scale to provide a more accurate measuring toward reliability of our current results.

## Conclusion

Drawing on social identity theory, this paper examines the effect of team resilience on team creativity through a sequential mediating mechanism. This study finds a positive relationship between team resilience and team creativity. Moreover, the empirical findings confirm the sequential mediation effect of team creative efficacy and team trust. That is, team resilience exerts a positive influence on team creativity through enhancing team creative efficacy and then increasing team trust. These results contribute to the development of linking resilience and creativity at the team level among undergraduate students through exploring the sequential mediators of different psychological characteristics.

## Data Availability Statement

The raw data supporting the conclusions of this article will be made available by the authors, without undue reservation.

## Ethics Statement

The studies involving human participants were reviewed and approved by the Vrije Universiteit Amsterdam Ethics Committee. The patients/participants provided their written informed consent to participate in this study.

## Author Contributions

MF and WC: conceptualization. WC and LJ: methodology, software, formal analysis, data curation, and writing—review and editing. MF, WC, and LJ: validation and writing—original draft preparation. WC: investigation and supervision. MF: resources, project administration, and funding acquisition. All authors have read and agreed to the published version of the manuscript.

## Conflict of Interest

The authors declare that the research was conducted in the absence of any commercial or financial relationships that could be construed as a potential conflict of interest.
